# Neurodevelopmental outcomes after moderate to severe neonatal hypoglycemia

**DOI:** 10.1007/s00431-020-03729-x

**Published:** 2020-07-14

**Authors:** Annett Helleskov Rasmussen, Sonja Wehberg, Fani Pørtner, Anna-Marie Larsen, Karen Filipsen, Henrik Thybo Christesen

**Affiliations:** 1 Hans Christian Andersen Children’s Hospital, Odense University Hospital, J.B. Windsloews Vej 4, Odense C, 5000 Odense, Denmark; 2 Institute of Clinical Research, Faculty of Health Sciences, University of Southern Denmark, Odense, Denmark; 3 Research Unit for General Practice, Department of Public Health, University of Southern Denmark, Odense, Denmark; 4 Department of Rehabilitation, Odense University Hospital, Odense, Denmark; 5 Odense Pancreas Center OPAC, Odense University Hospital, Odense, Denmark

**Keywords:** Neonatal hypoglycemia, Cerebral impairment, Follow-up, Risk groups

## Abstract

The long-term consequences of transient neonatal hypoglycemia are sparsely studied. We performed a follow-up of a cohort of neonates with blood glucose recordings < 1.7 mmol/L (< 30 mg/dL), treated with > 2.5 mmol/L (> 45 mg/dL), compared with healthy siblings. Exclusion criteria were gestational age < 35 weeks, severe asphyxia, head injury, and other cerebral diseases. In 71 children with neonatal hypoglycemia and 32 control siblings, Wechsler IV cognitive test, Movement ABC-2 test, and Child Behavior Checklist were performed at mean age 7.75 and 9.17 years, respectively. No significant changes were detected for cognitive function by using Wechsler IV or for behavior by using Child Behavior Checklist. In univariate analysis, the hypoglycemia group had lower age-adjusted fine motor scores by using the Movement ABC-2 test compared with control siblings, 42.6 ± 31.2 vs. 57.2 ± 30.8 percentile (*p* = 0.03). In the sibling-paired analysis, the decrease in total motor score was highly significant, *p* = 0.009, driven by a decrease in fine motor score, *p* = 0.008. In the hypoglycemia group, adjusted analysis showed a lower fine motor function for boys, *β* = − 16.4, *p* = 0.048.

*Conclusion*: Neonatal hypoglycemia treated with > 2.5 mmol/L was associated with lower fine motor scores within the normal range, particularly in boys. No associations with cognitive function or behavior were detected.

**Table:** 

**What is Known:** • *Transient neonatal hypoglycemia is associated with acute neurologic dysfunction and long-term neurodevelopment impairment in 18 months of age.*	
**What is New:** • *Neonatal hypoglycemia treated with > 2.5 mmol/L is associated with lower fine motor function within the normal range, particularly in boys, but not to changes in cognitive function or behavior.*

**What is Known:**

• *Transient neonatal hypoglycemia is associated with acute neurologic dysfunction and long-term neurodevelopment impairment in 18 months of age.*

**What is New:**

• *Neonatal hypoglycemia treated with > 2.5 mmol/L is associated with lower fine motor function within the normal range, particularly in boys, but not to changes in cognitive function or behavior.*

Transitional low plasma glucose concentration is a physiological event during the first 1–3 days of life in term-born neonates, but in risk groups may be more prolonged and more severe [1, 2]. Hypoglycemia is associated with acute neurologic dysfunction and long-term neurodevelopment impairment in a small but significant group of neonates [3–5].

There is no universal consensus on a “safe” blood glucose level for newborn infants, partly because individual susceptibility to brain injury varies with factors such as gestational age (GA), the presence of comorbid conditions, and the ability of the infant to produce and use alternative cerebral fuels [6]. Risk groups for deeper and/or prolonged hypoglycemia are defined by conditions with small glycogen stores, e.g., prematurity, intrauterine growth retardation, small for gestational age (SGA), dysmaturity and low birth weight, or conditions with high glucose utilization including infants of mothers with diabetes, asphyxia, and septicemia. Large for gestational age (LGA) is often considered to be an independent risk factor, although this in some cases may represent undiscovered gestational diabetes.

Impaired cerebral outcome in neonates at risk of hypoglycemia may be caused not only by hypoglycemia but also by the underlying risk factor. In addition, socioeconomic factors have been associated with neurodevelopmental performance [1, 7–16]. Most guidelines focus to prevent and treat hypoglycemia in neonates at risk, mainly aiming to keep blood glucose above 2.5 mmol/L (45 mg/dL) after the physiological nadir in the first hours of life [11, 12, 17]. In a recent randomized controlled trial (RCT) in at-risk neonates ≥ 35 weeks without hypoglycemic symptoms, no difference in neurodevelopmental outcome was observed at 18 months between groups with an intervention threshold < 2.0 mmol/L vs. < 2.6 mmol/L [18]. On the other hand, at-risk neonates with glucose values < 2.0 mmol/L had lower visual-motor and executive function at 4.5-year follow-up compared with at-risk neonates with normoglycemia [19].

We aimed to evaluate the impact of transient moderate or severe neonatal hypoglycemia with a longer follow-up to 6–9 years of age with determination of cognitive, motor, and behavioral scores.

## Patients and methods

Our observational follow-up study was based on a cohort of neonates admitted to the neonatal ward at Hans Christian Andersen Children’s Hospital at Odense University Hospital from August 1, 2004 to August 1, 2008. The hospital is a tertiary regional public hospital with approximately 4500 births/year. At the time of the inclusion period, children at risk of hypoglycemia had early feeding and supplemental feeds to prevent hypoglycemia, were monitored with repeat blood glucose samplings, and were treated aiming to maintain blood glucose above 2.5 mmol/L by oral feeds or i.v. glucose.

Hospital files with any diagnosis of neonatal hypoglycemia (WHO IDC10 diagnosis codes DP 70.0-70.9) were retrospectively retrieved for validation of the hypoglycemia diagnosis, blood glucose recordings, inclusion/exclusion criteria, and hypoglycemia risk factors.

Study inclusion criteria were one or more episodes of moderate or severe hypoglycemia, defined as blood glucose before 2 h of age between 0.5 and 1.0 mmol/L (0–18 mg/dL; moderate), or below 0.5 mmol/L (9 mg/dL; severe); and from 2 h onwards 1.0–1.6 mmol/L (18–29 mg/dL; moderate), or below 1.0 mmol/L (severe). Exclusion criteria were 1) GA < 35 + 0 weeks + days, 2) severe asphyxia (cord pH < 7.0, base excess < − 15.0, or Apgar score 0–3/1 min), and 3) hospitalizations until follow-up for head injury, meningitis, or any other known potential cerebral damaging condition. These exclusion criteria were chosen to reduce the impact of comorbid conditions on the cerebral outcome.

From the hospital files, the following data were extracted: GA (weeks + days), birth weight (g), umbilical cord blood pH and base excess (mmol/L), Apgar score at 1 and 5 min, maternal diabetes, and number of recorded moderate-severe hypoglycemic episodes. Hypoglycemia risk groups were defined as follows: maternal diabetes (any kind), mild-moderate asphyxia (cord pH 7.0–7.1 or base excess − 10 to − 15), late preterm birth (GA 35 + 0 to 36 + 6), and SGA and LGA (birth weight exceeding ± 2 SD). The diagnoses of maternal diabetes, asphyxia, preterm birth, SGA, and LGA were validated from the files and hence not only based on discharge diagnosis codes. We did not have permission to access the mothers’ files to check missing information on gestational diabetes in the neonatal files.

Follow-up examinations were performed at child age 6–9 years. As an internal control, all healthy siblings aged 3–16 years without any hospital records of neonatal hypoglycemia and without any exclusion criteria were invited to participate in the same setup. Healthy siblings were chosen as controls to minimize genetic and environmental factors. No participant in the hypoglycemia group had more than one eligible sibling, and no eligible siblings declined to participate in the study. Age and sex differences were overcome by formal testing with results expressed in age- and sex-depending percentiles according to normative population data. To be included as sibling, a minimum of one test should be performed, allowing inclusion if another test could not be performed in case of inappropriate low age or difficulties with corporation.

### Measures and procedures

Cognitive function was evaluated using Wechsler’s intelligence scale for children fourth edition (WISC-IV) [13]. Motor function was evaluated using Movement Assessment Battery for Children 2 (Movement ABC-2) [20]. Behavior was evaluated using the parental questionnaire Achenbach Child Behavior Checklist (CBCL) [14, 15]. A single pediatric psychologist with experience in examining children conducted the WISC-IV testing and scoring. The examiner was blinded with regard to all clinical data and participant group. The participants were excluded from WISC-IV, if they had performed a similar test before, but still, they participated in the remaining test program. Two physiotherapists with expertise in children conducted the Movement ABC-2 testing and did the final scoring. The examiners were likewise blinded.

### Statistical methods

The association between neonatal hypoglycemia and outcomes was evaluated by stratifying for hypoglycemia severity by lowest recorded blood glucose value. Comparisons between groups were performed using the Student *T* test, or *χ*^2^ test/Fisher’s exact test where appropriate. Paired *t* test was used to compare siblings.

For all children, the outcome measures were analyzed for the effect of the following explanatory factors in regression models: maternal, paternal and highest parental education, breastfeeding, hypoglycemia, and child’s sex. We accounted for clustering due to siblings in our linear regression analysis. In multivariable analysis for all children, we included variables with a univariate *p* value < 0.10 for any outcome and backwards-eliminated variables with the highest *p* value. The final model included hypoglycemia, mothers’ education level, and child sex, applied on all children and split on the hypoglycemia group and the control group. Statistical significance was assumed for a *p* value < 0.05. Data were analyzed using Stata (StataCorp, College Station, TX, USA).

IQ score by using WISC-IV was a priori chosen as the primary outcome. A power calculation (alpha 0.05, beta 1–0.80, *n* = 103, WISC-IV SD = 15 points, *n* = 103) showed that our study was powered to detect a true difference in IQ score of 8.4 points or above.

## Results

**Fig. 1:**
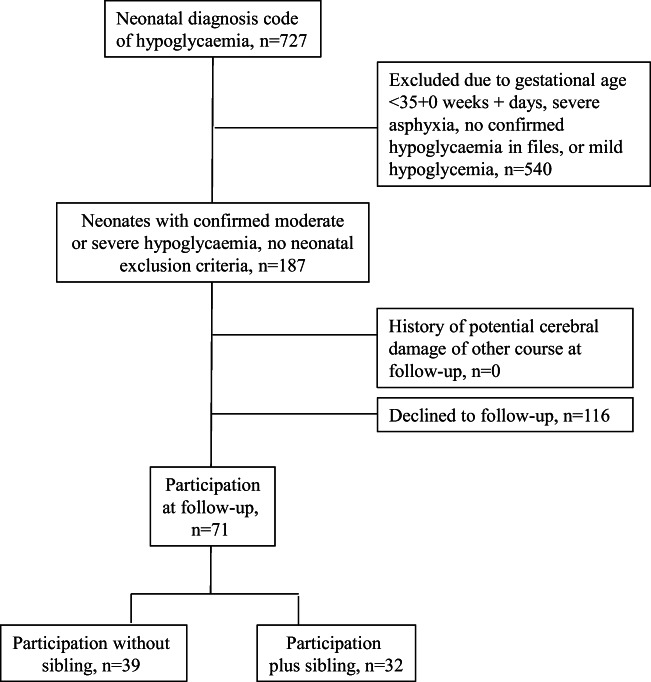
Participants inclusion flowchart

The study included 103 children, of which 71 (37% girls, *n* = 26 and 63% boys, *n* = 45) had neonatal hypoglycemia and follow-up investigations (Fig. 1). The predominant hypoglycemia risk groups were maternal diabetes (*n* = 16) and SGA (*n* = 17), followed by prematurity, asphyxia, and LGA (*n* = 8, 5, and 4, respectively). Twenty-one neonates had no identifiable risk group. None of the included patients had seizures, encephalopathy, or a later diagnosis of hormonal or metabolic disease, and none was treated with other medication than i.v. glucose. The median (range) age at follow-up was 7.75 (6.0–8.45) years.

The internal control group consisted of 32 healthy siblings (43% girls and 57% boys) without neonatal hypoglycemia and no other hypoglycemia risk group assignment at a median age of 9.17 (3.75–16.0) years. The neonatal characteristics of children with and without hypoglycemia are listed in Table 1. The outcome scores at follow-up for WISC-IV, Movement ABC-2, and CBCL are presented in Table 2.

**Table 1:** Neonatal characteristics of 103 children with and without hypoglycemia

	Hypoglycemia group *N* = 71	Control siblings *N* = 32	*p* value	Hypoglycemia group		*p* value
				Moderate* hypoglycemia	Severe** hypoglycemia	
Girls/boys (*n*/*n*)	26/45	15/17	0.32	9/10	18/35	0.43
Gestational age (weeks + days)	38 ± 2.1	38 ± 1.6	0.94	39 ± 1.9	38 ± 2.6	0.64
Base excess (mmol/L)	− 4.1 ± 4.6	− 3.4 ± 4.8	0.40	− 4.4 ± 4.9	− 3.3 ± 4.9	0.38
Cord pH	7.2 ± 0.1	7.2 ± 0.1	0.89	7.2 ± 0.1	7.3 ± 0.1	0.56
Birth weight (g)	3178 ± 898	3439 ± 638	0.20	3234 ± 912	3085 ± 822	0.48
Apgar at 1 min	8.8 ± 1.5	9.0 ± 1.2	0.57	8.6 ± 1.6	8.9 ± 1.5	0.45
Apgar at 5 min	9.7 ± 0.6	9.0 ± 0.6	0.33	9.7 ± 0.7	9.6 ± 0.7	0.83
Hypoglycemic episodes (*n*)	1.7 ± 1.0	0	-	1.5 ± 1.0	2.4 ± 1.6	*0.001*

Values are in mean ± 1SD if not otherwise stated. Significant *p* values are italicized

*Age < 2 h, blood glucose 0.5–1.0 mmol/L; age ≥ 2 h, blood glucose 1.0–1.6 mmol/L

**Age < 2 h, blood glucose < 0.5 mmol/L; age ≥ 2 h, blood glucose < 1.0 mmol/L. All values except child sex are expressed as mean ± SD

**Table 2:** Cognitive, motor, and behavioral outcomes for neonates with hypoglycemia and their control siblings

	Hypoglycemia (*N* = 71)	Controls (*N* = 32)		Hypoglycemia patients matched 1:1 with control siblings			Hypoglycemia severity		
				Hypoglycemia	Control siblings		Moderate*	Severe**	
	Mean (95% CI)	Mean (95% CI)	*p* value	Mean (95% CI)	Mean (95% CI)	*p* value	Mean (95% CI)	Mean (95% CI)	*p* value
Intelligence quotient									
WISC-IV	*N* = 71	*N* = 26		*N* = 23	*N* = 23		*N* = 53	*N* = 18	
Total score	96.8 (93.3–99.8)	99.7 (95.0–104.4)	0.30	97.3 (91.1–102.8)	99.3 (104.6)	0.20	96.4 (92.9–99.9)	99.0 (92.0–104.0)	0.64
Verbal comprehension	99.1 (96.1–102.2)	102.7 (97.4–108.0)	0.23	100.6 (94.3–106.0)	102.2 (96.2–108.1)	0.52	99.0 (95.6–102.4)	99.4 (92.2–106.6)	0.92
Perceptual organization	100.9 (97.8–104.0)	104.7 (101.0–108.5)	0.16	100.5 (94.7–101.3)	104.8 (100.6–109.1)	0.12	100.8 (97.1–104.4)	101.3 (94.5–108.0)	0.88
Processing speed	101.2 (97.5–105.0)	99.6 (93.9–106.0)	0.71	97.1 (90.6–103.7)	99.5 (92.5–106.6)	0.45	99.7 (96.0–103.8)	105.5 (97.0–114.1)	0.16
Working memory	89.9 (86.5–93.4)	92.6 (87.8–98.1)	0.34	91.6 (84.5–98.7)	92.7 (87.0–98.4)	0.75	89.4 (85.5–94.3)	89.4 (84.5–95.4)	0.99
Motor function									
Movement ABC	*N* = 68	*N* = 29		*N* = 23	*N* = 23		*N* = 47	*N* = 18	
Total motor	48.3 (40.5–72.4)	60.7 (49.1–72.4)	*0.07*	42.6 (29.9–55.3)	60.8 (48.4–73.2)	*0.009*	48.0 (38.4–57.8)	49.2 (35.1–63.3)	0.89
Fine motor	42.6 (34.8–50.3)	57.2 (45.6–68.7)	*0.03*	40.4 (26.9–53.9)	55.7 (43.6–67.8)	*0.008*	43.3 (34.0–52.6)	40.4 (25.2–55.5)	0.73
Gross motor	49.0 (42.0–56.1)	53.0 (42.2–56.1)	0.53	40.7 (40.3–63.7)	52.1 (40.3–63.9)	*0.06*	49.3 (41.0–57.7)	48.2 (34.0–62.4)	0.88
Balance	58.8 (51.6–66.0)	64.0 (53.0–75.2)	0.43	58.7 (47.4–70.1)	58.7 (51.9–76.3)	0.53	57.1 (48.5–65.7)	63.8 (49.7–77.9)	0.41
Behavior									
CBCL (T-scores)	*N* = 47	*N* = 9		*N* = 9	*N* = 9		*N* = 34	*N* = 13	
Internalizing	54.1 (46.1–63.1)	61.5 (54.6–67.8)	0.39	54.2 (46.0–63.1)	56.9 (46.1–67.9)	0.65	62.4 (54.3–71.3)	56.2 (47.6–65.6)	0.39
Externalizing	52.2 (48.7–55.9)	50.1 (41.2–60.0)	0.70	49.5 (41.9–57.1)	50.6 (41.2–60.0)	0.66	53.1 (48.8–58.0)	42.1 (43.7–55.0)	0.31

Values are in mean (95% CI) if not otherwise stated. Significant and trend *p* values are italicized

*WISC-IV*, Wechsler’s intelligence scale for children, fourth edition; *CBCL*, Child Behavior Checklist

*Age < 2 h, blood glucose 0.5–1.0; age > 2 h, blood glucose 1.0–1.6

**Age < 2 h, blood glucose < 0.5; age > 2 h blood glucose < 1.0

### WISC-IV associations

In the hypoglycemia group, five (7%) had a full-scale IQ in the range 71–79, which denotes to below average. They all had moderate hypoglycemia, none had known risk factor, and none of them had siblings enrolled. In contrast, none of the siblings had an IQ below average. The mean (95% CI) full-scale IQ score was 96.8 (93.3–99.8) in the hypoglycemia group. Although this IQ score was 3.2 points lower compared with the normative population, no significant differences were found from sibling controls, neither when comparing paired siblings. No difference in full IQ was observed between those with moderate vs. severe hypoglycemia, and no differences were seen in any of the WISC-IV subtests between the groups.

We analyzed whether the individual hypoglycemia risk factors affected the outcomes differentially for children with neonatal hypoglycemia. No differences in IQ score were found when comparing the hypoglycemia risk groups (each or pooled) with siblings, or children with hypoglycemia with and without an identified hypoglycemia risk (Table 3 and Table 4).

**Table 3:** Univariable analysis of associations between the hypoglycemia group and siblings, overall and by risk groups

	Total IQ score		Total motor function		Fine motor function		CBCL internalizing	
	*β* (95% CI)*	*p* value	β (95% CI)*	*p* value	*β* (95% CI)*	*p* value	*β* (95% CI)*	*p* value
Siblings, baseline	99.7 (95.0; 104.4)		60.7 (49.2; 72.5)		57.2 (45.5; 68.1)		54.7 (30.6; 86.9)	
Any risk group		0.86		0.30		0.34		0.42
Maternal diabetes	− 3.8 (− 11.8; 4.2)	0.35	2.1 (− 19.1; 23.4)	0.83	− 4.0(− 25.4; 17.6)	0.70	3.9 (− 15.3; 23.9)	0.69
Asphyxia	− 2.4 (− 14.6; 9.5)	0.67	− 16.5 (− 19.2; 23.5)	0.28	− 25.7 (− 55.3; 4.2)	0.10	5.6 (− 19.7; 31.8)	0.35
Premature	0.1 (− 10.1, 9.8)	0.98	− 11.9 (− 38.0; 14.3)	0.37	− 11.7 (− 37.2; 14.8)	0.37	1.8 (− 26.1; 30.5)	0.89
SGA	− 3.2 (− 11.3; 4.9)	0.42	− 11.2 (− 31.2; 7.4)	0.22	− 14.5 (− 33.4; 4.6)	0.13	19.0 (1.5; 38.4)	*0.04*
LGA	− 9.7 (− 24.8; 5.3)	0.20	− 11.8 (−44.3; 21.9)	0.50	− 22.4 (− 55.8; 10.4)	0.18	− 8.5 (− 24.5; 32.2)	0.55
No identified risk group	− 2.2 (− 9.4; 4.9)	0.53	− 20.9 (− 38.9; − 2.9)	*0.02*	− 17.6 (− 35.4; 0.2)	*0.05*	1.8 (− 15.7; 19.4)	0.83

*Except for baseline values, significant values are italicized

*SGA*, small for gestational age; *LGA*, Large for gestational age

**Table 4:** Univariable analysis between participant characteristics and outcomes for all children

	Total IQ score		Total motor function		Fine motor function		CBCL internalizing		CBCL externalizing	
	*ß* (95% CI)*	*p* value	*ß* (95% CI)*	*p* value	*ß* (95% CI)*	*p* value	*ß* (95% CI)*	*p* value	*ß* (95% CI)*	*p* value
Mother’s education		*0.02*		*0.10*		0.31		0.71		0.23
High school (baseline)	101.3 (86.8; 120.3)		57.3 (26.7; 87.9)		45.0 (14.0; 79.9)		45.5 (20.5; 70.4)		46.6 (32.8; 60.4)	
1–2 years after high school	− 5.4 (− 19.2; 8.9)	0.43	− 3.4 (− 36.2; 29.3)	0.83	3.6 (− 29.6; 36.7)	0.82	14.8 (− 11.9; 41.5)	0.27	4.0 (− 10.8; 18.8)	0.58
3–4 years after high school	− 6.5 (− 20.2; 7.6)	0.36	− 16.2 (− 49.5; 17.9)	0.33	− 6.8 (− 40.4; 26.1)	0.69	17.4 (− 9.7; 44.4)	0.20	6.3 (− 8.7; 21.3)	0.40
5–7 years after high school	7.2 (− 7.1; 22.5)	0.29	18.6 (− 18.9; 56.6)	0.32	27.3 (− 10.4; 65.9)	0.16	11.2 (− 17.7; 39.9)	0.44	2.1 (− 13.7; 18.2)	0.78
Not answered	− 4.3 (− 18.4; 9.5)	0.50	− 5.3 (− 37.2; 27.9)	0.75	1.0 (− 31.7; 33.8)	0.95	18.8 (− 11.7; 49.5)	0.22	15.2 (− 1.1; 32.2)	*0.07*
Father’s education		0.69		0.78		0.43		0.89		0.22
High school (baseline)	96.2 (89.5; 103.4)		57.3 (26.6; 87.6)		48.6 (31.6; 66.1)		59.6 (45.7; 73.4)		49.8 (42.2; 57.3)	
1–2 years after high school	0.4 (− 9.2; 10.1)	0.93	− 3.4 (− 36.2; 29.9)	0.98	3.2 (− 22.6; 28.8)	0.80	5.5 (− 14.3; 25.6)	0.58	3.2 (−7.7; 14.5)	0.55
3–4 years after high school	1.4 (− 7.3; 10.6)	0.75	− 0.4 (− 21.9; 26.8)	0.99	− 7.9 (− 29.7; 13.3)	0.45	− 0.6 (− 17.3; 16.0)	0.94	2.0 (− 7.1; 11.5)	0.65
5–7 years after high school	6.7 (− 3.8; 17.2)	0.21	152 (− 12.1; 47.3)	0.27	14.7 (− 12.3; 42.0)	0.27	− 3.7 (− 23.7; 16.3)	0.71	−2 .4 (− 13.4; 8.4)	0.65
Not answered	0.7 (− 7.7; 9.2)	0.86	1.8 (− 18.3; 22.5)	0.86	− 2.6 (− 23.2; 18.2)	0.80	4.8 (− 17.7; 27.3)	0.67	12.1 (− 0–2; 24.4)	*0.06*
Highest education		*0.03*		*0.08*		0.12		0.68		0.43
High school (baseline)	108.0 (93.9–129.2)		51.3 (16.5–86.6)		47.6 (11.9–83.9)		48.8 (18.3–79.3)		48.0 (30.8–65.2)	
1–2 years after high school	− 13.2 (− 31.3; 4.6)	0.12	− 1.2 (− 38.2; 40.8)	0.95	1.7 (− 38.9; 41.5)	0.93	18.1 (− 15.1; 51.3)	0.27	4.0 (− 14.7; 22.8)	0.67
3–4 years after high school	− 13.5 (− 30.3; 3.7)	0.11	− 8.9 (− 45.7; 27.7)	0.63	− 9.9 (− 47.1; 27.0)	0.59	10.3 (− 21.5; 42.1)	0.51	3.8 (− 14.1; 21.8)	0.67
5–7 years after high school	− 2.5 (− 20.1; 15.0)	0.78	20.4 (− 18.5; 59.8)	0.30	18.0 (− 21.9; 57.8)	0.36	7.4 (− 25.4; 40.2)	0.65	0.8 (− 17.6; 19.4)	0.92
Not answered	− 10.4 (− 27.7; 6.8)	0.22	2.2 (− 34.4; 38.1)	0.90	0.16 (− 36.4; 37.2)	0.99	14.4 (− 20.1; 49.1)	0.41	11.6 (− 7.9; 31.2)	0.23
Breastfeeding										
No (baseline)	98.9 (95.1; 104.8)		54.8 (46.1; 63.4)		48.6 (40.2; 56.4)		56.2 (45.9; 66.5)		53.9 (47.9; 59.8)	
Yes	− 1.5 (− 6.7;3.6)	0.56	− 6.5 (− 18.8; 6.6)	0.32	− 3.6 (− 16.2; 9.6)	0.58	5.6 (− 6.7; 17.9)	0.37	− 2.6 (− 9.7; 4.4)	0.45
Sex										
Female (baseline)	99.0 (95.5; 106.2)		62.2 (51.3; 72.1)		57.6 (47.0; 67.5)		66.8 (58.8; 74.9)		51.9 (47.2; 56.8)	
Male	− 2.4 (− 7.4; 7.6)	0.34	− 15.8 (− 28.1; − 2.7)	*0.018*	− 17.1 (− 29.2; − 4.0)	*0.01*	− 12.6 (− 23.6; − 1.6)	*0.03*	0.1 (− 6.4; 6.7)	0.97
Hypoglycemia										
No (baseline)	99.7 (95.0; 104.4)		60.7 (− 26.2; 1.4)		57.2 (45.9; 68.4)		54.6 (40.2; 68.8)		50.1 (42.4; 58.7)	
Yes	− 2.9 (− 8.5; 2.6)	0.30	− 12.4 (− 26.1; − 1.4)	*0.08*	− 14.6 (− 28.2; − 0.9)	*0.03*	6.5 (− 8.9; 22.1)	0.39	1.7 (− 7.2; 10.6)	0.70
Risk group										
No risk group (baseline)	98 (95; 102)		52 (43; 61)		49.9 (41.1; 58.8)		55.7 (47.1; 64.4)		51.0 (46.0; 55.9)	
All risk groups	− 2 (− 7; 3)	0.37	0 (− 13.0; 13.1)	0.99	− 5.9 (− 18.9; 7.0)	0.36	7.4 (− 3.7; 19.0)	0.18	1.7 (− 4.8; 8.4)	0.59

*Except for baseline values, significant and trend *p* values are italicized

We further analyzed whether hypoglycemia and other baseline parameters associated with the outcomes for the total of the children with hypoglycemia and siblings. In the univariable regressions, the level of mother’s education affected the mean total IQ score (test for trend *p* = 0.02) in a seemingly U-shaped pattern (Table 4). No associations were seen on father’s educational level, breastfeeding, child sex, or hypoglycemia status. In the multivariable regression analysis, the mother’s educational level associated likewise with the total IQ score, which is the only significant association (Table 5). This association was driven by a trend in the hypoglycemia group (*p* = 0.09).

**Table 5:** Multivariable analyses for specific parameters for all children with and without hypoglycemia

	Total IQ score		Total motor function		Fine motor function		CBCL internalizing	
	Adjusted *ß* (95% CI)*	*p* value	Adjusted *ß* (95% CI)*	*p* value	Adjusted *ß* (95% CI)*	*p* value	Adjusted *ß* (95% CI)ss	*p* value
A: All children								
Mother’s education		*0.03*		0.19		0.26		0.03
High school (baseline)	103.6 (89.3; 118.3)		72.8 (39.6; 105.9)		62.9 (29.8; 96.1)		46.1 (16.7; 75.6)	
Plus 1–2 years	− 6.1 (− 20.1; 8.3)	0.38	− 5.4 (− 37.8; 27.1)	0.74	0.9 (− 31.4; 33.3)	0.95	14.3 (− 11.3; 39.9)	0.26
Plus 3–4 years	− 7.0 (− 21.4; 7.7)	0.33	− 16.6 (− 49.4; 16.1)	0.31	− 7.5 (− 40.3; 25.2)	0.65	17.3 (− 8.7; 43.3)	0.18
Plus 5–7 years	7.1 (− 8.1; 22.6)	0.36	13.5 (− 23.4; 50.6)	0.46	21.4 (− 15.6; 58.5)	0.25	9.4 (− 18.9; 37.8)	0.51
Not answered	− 5.0 (− 18.8; 9.5)	0.47	− 6.1 (− 38.4; 26.3)	0.71	− 0.8 (− 33.2; 31.5)	0.96	19.4 (− 10.4; 49.2)	0.19
Male sex	− 1.1(− 6.0; 8.3)	0.66	− 12.4 (− 25.7; 0.7)	*0.06*	− 13.2 (− 26.2; − 0.2)	*0.04*	**−** 13.4(− 24.8; -2.1)	*0.02*
Hypoglycemia	− 2.2 (− 7.5; 3.3)	0.41	− 9.4 (− 23.2; 4.4)	0.18	− 11.3 (− 25.1; 2.3)	*0.10*	8.3(− 7.4; 24.1)	0.3
B: Hypoglycemia								
Mother’s education		*0.09*		0.34		0.20		0.78
High school (baseline)	101.3 (87.4; 115.2)		65.2 (19.1; 91.6)		53.1 (17.5; 88.6)		53.6 (− 27.3; − 0.46)	
Plus 1–2 years	− 6.5 (− 21.3; 8.4)	0.39	− 4.2 (− 37.2; 29.2)	0.80	0.4 (− 33.7; 34.6)	0.98	16.9 (− 9.6; 48.2)	0.24
Plus 3–4 years	− 7.3 (− 22.4; 7.6)	0.33	− 16.8 (− 50.2; 16.1)	0.32	− 7.9 (− 41.6; 25.7)	0.63	19.3 (− 9.6; 48.2)	0.18
Plus 5–7 years	8.1 (− 8.7; 25.0)	0.34	14.4 (− 26.3; 55.1)	0.48	29.9 (− 10.2; 70.2)	0.14	12.7 (− 19.6; 44.2)	0.42
Not answered	− 4.9 (− 19.8; 9.8)	0.50	− 5.3 (− 39.8; 28.1)	0.75	− 0.3 (− 33.9; 33.2)	0.98	19.7 (− 14.9; 53.2)	0.25
Male sex	− 0.1 (− 6.1; 6.2)	0.97	− 15.9 (− 32.2; 1.7)	*0.065*	− 16.4 (− 32.8; 0.13)	*0.048*	− 13.7 (− 27.6–0.46)	*0.044*
C: Healthy siblings								
Mother’s education		0.41		0.66		0.92		0.13
High school (baseline)	100.5 (− 13.9; 5.7)		64.3 (41.6; 86.9)		60.0 (30.1; 18.9)		60.2 (41.7; 78.6)	
Plus 1–2 years	-	-	-	-	-	-	-	-
Plus 3–4 years	− 0.4 (− 12.1; 11.2)	0.93	− 1.1 (− 29.9; 27.7)	0.93	3.1 (− 25.9; 32.2)	0.82	8.1 (− 27.3; 20.9)	0.41
Plus 5–7 years	0.6 (− 13.9; 15.3)	0.92	− 11.8 (− 47.3; 23.8)	0.50	− 7.7 (− 43.9–28.8)	0.67	− 3.2 (− 27.3; 20.9)	0.73
Not answered	-	-	18.0 (− 24.3; 60.3)	0.39	-	-	− 14.4 (− 23.6; 5.8)	0.12
Male sex	− 4.1 (− 13.9; 5.7)	0.40	− 5.9 (− 31.2–19.3)	0.63	− 6.5 (− 32.2; 18.9)	0.56	− 4.2 (− 24.4–16.1)	0.57

*Except for baseline values, significant and trend *p* values are italicized

### Movement ABC-2 associations

In the Movement ABC-2 test, full-scale motor impairment defined as score < 15th percentile was seen in 9 in the hypoglycemia group and in 2 controls. Subtests scores below the 8th percentile were only seen in the hypoglycemia group. These differences did not reach significance. The hypoglycemia group had a lower fine motor score compared with controls, 42.6 ± 31.2 vs. 57.2 ± 30.8 percentile, *p* = 0.03 (Table 2). No differences were seen in gross motor score or balance.

In the sibling-paired analysis, the decrease in total motor score was highly significant, *p* = 0.009, driven by a decrease in fine motor score, *p* = 0.008. No differences were seen between the moderate vs. severe hypoglycemia groups. Within the hypoglycemia group, no differences in total or fine motor score were found for the individual hypoglycemia risk factor compared with siblings (Table 3).

When pooling all participants in search for univariable associations, an impression of a U-shaped relation between maternal educational level and offspring total motor function was seen (Table 4). Boys had lower total and fine motor scores compared with girls. Hypoglycemia was associated with a significantly lower fine motor score. No associations were seen on breastfeeding.

Multivariable regression analysis showed a reduced fine motor function in boys, *β* = − 13.2, *p* = 0.04, but no independent association with hypoglycemia (Table 5). Test for interaction between sex and group by hypoglycemia was non-significant. In the adjusted subgroup analysis, the fine motor function was significantly lower for boys in the hypoglycemia group, *β* = − 16.4, *p* = 0.048, with no impact of sex in the control group.

### CBCL internalizing associations

In the child mental health test CBCL, no differences were observed between the hypoglycemia group and the control group, in the sibling-paired analysis, or when comparing moderate vs. severe hypoglycemia (Table 2). An internalizing T-score denoting borderline (60–70), or pathologic (> 70) score, was seen in six, and twelve, children in the hypoglycemia group vs. three, and one, in controls (non-significant differences).

The CBCL score did not differ between neonatal risk factor groups, except for hypoglycemic neonates with SGA, who had a higher internalizing score (Table 3). No difference was seen between those with an identified risk group compared with no risk assignment. No associations were found on parental education, breastfeeding, or hypoglycemia in the analysis of all participants (Table 4).

## Discussion

In this 6–9-year follow-up of children with moderate-severe neonatal hypoglycemia and their healthy siblings, no differences in IQ score, motor function scores, or behavioral scores were seen between the groups in adjusted analyses. Among the children with neonatal hypoglycemia, boys had significantly lower fine motor score and girls had higher CBCL internalizing scores in adjusted analyses, however, within the normal range. Neonatal hypoglycemia was not associated with an increased risk of neurodevelopmental impairment or borderline/pathological internalizing score.

Many observational studies have demonstrated neurodevelopmental impairment and/or abnormal MRI scan of the brain after neonatal hypoglycemia, depending on the severity, duration, and comorbid factors [3, 16, 23–25]. High-evidence clinical studies on the cerebral outcome after neonatal transient hypoglycemia are sparse. In the recent Dutch RCT, no differences were seen in at-risk neonates randomized to intervention threshold < 2.0 mmol/L vs. < 2.6 mmol/L at 18 months of follow-up [18]. However, the two groups had similar mean glucose values during the first 2 days (3.2 vs. 3.4 mmol/L), and the follow-up time was relatively short. The low-threshold group had more episodes of recurrent and of severe hypoglycemia, which impact may only become overt after a longer follow-up time.

The importance of longer follow-up time was overt in the hypoglycemia studies from New Zealand. In an RCT, oral dextrose was more successful in preventing repeat hypoglycemia than placebo [21], but no difference in adverse neurodevelopmental outcome defined as scores < 1 SD, blindness, deafness or cerebral palsy were found at 2 years of follow-up [22]. From the same hospital’s background cohort of 528 at-risk neonates (primarily GA 35 + 0 to 36 + 6 weeks, weight < 10 lb or > 90 lb for GA, or maternal diabetes), the neurodevelopmental outcome at the age of 2 years was comparable between those with hypoglycemia (< 2.6 mmol/L) and those without [23]. At 4.5 years of follow-up, the combined neurosensory impairment outcome was likewise not associated with hypoglycemia, but impaired executive and visual-motor functions were observed with especially increased risk for those with severe (< 2.0 mmol/L), recurrent, or clinically undetected episodes [19]. These findings gave concern of impaired later learning capability.

Our study was smaller, but with a longer follow-up time and with an inclusion criteria of more severe neonatal hypoglycemia (< 1.7 mmol/L after 2 h). In both the New Zealand study and our study, the neonates were treated to avoid repeat blood glucoses below 2.5–2.6 mmol/L, and the risk groups were largely comparable. Our data supports the conclusion from the New Zealand cohort that short-lasting neonatal hypoglycemia does not result in significant adverse neurodevelopmental outcome.

### Hypoglycemia risk factors

Our data did not support that any of the individual hypoglycemia risk groups, or the pooled group of children with a risk hypoglycemia factor, had adverse cerebral outcome. In contrast, McKinlay et al. [23] detected adverse neurological outcome (< 1 SD) in 42% of the at-risk children with or without blood glucose below 2.6 mmol/L. Interestingly, the authors also found that those with maximal interstitial glucose concentration by continuous monitoring above the median had a higher risk of adverse neurological outcome.

Adverse adjusted associations with neonatal hypoglycemia < 1.7 mmol/L have been detected in neonates with more profound perinatal risk, e.g., GA < 35 weeks. Kerstjens et al. [16] showed significantly increased adjusted odds ratio for developmental delay for late preterms (GA 32 + 0 to 35 + 6 weeks) with blood glucose < 1.1 mmol/L by using the parental Ages and Stages Questionnaire (ASQ) at 46 months of age. No significant associations were seen on specific ASQ domains (fine or gross motor, communication, problem-solving, personal-social). We were not able to detect any trend with respect to hypoglycemia severity, which may be attributed to a more rapid correction of hypoglycemia, a higher GA, or lower study power.

In maternal diabetes, term neonates with hypoglycemia down to 0.0–1.5 mmol/L had adverse neurodevelopmental outcome for those aged 7–8 years compared with normoglycemic offspring and healthy controls, including Gilberg’s test for minimal brain damage, Movement ABC, and Griffith’s mental development scales [25]. More recently, Kaiser et al. found adjusted associations between early transient hypoglycemia (< 2.5 mmol/L < 3 h of age) and school performance at 10 years of follow-up in 1395 children with GA down to 23 weeks [26].

For the individual risk factors, we detected a univariate association between SGA and a higher CBCL internalizing score within the normal range. Others have shown psychological association in SGA children, including increased anxiety and depression in adults born SGA and with extremely/very low birth weight [27], and adjusted associations between SGA, but not GA, and lower ASQ scores at 46 months [16].

A relatively large part of our neonates had no identifiable risk factor. We assume that infrequent recording of maternal gestational diabetes status in the neonatal files was a dominant explanation, although children without any known risk factor also may develop hypoglycemia.

### Sex-differential vulnerability to hypoglycemia

The adjusted significant associations with lower fine motor score for boys within our hypoglycemia group suggested a sex-differential vulnerability to neonatal hypoglycemia. No differences were seen between child sexes for the healthy controls in accordance with the test protocols. Although the siblings group was smaller and potentially underpowered for statistical analysis, the beta coefficients for fine motor function and internalizing were less than half compared with the hypoglycemia group. Others have found adverse neurological outcome in boys with a perinatal risk factor. In late preterms, male sex independently associated with abnormal parental ASQ at 46-month-old late preterms, irrespective of hypoglycemia status [16].

Other late preterm studies support the susceptibility of males to adverse neurodevelopmental outcomes as reviewed by Baron et al. [28], but without data on hypoglycemia as the eventual additional risk factor and with no data on motor function and behavior. For maternal type 1 diabetes, male sex increases the risk of lower IQ in adult offspring in adjusted analysis, whereas neonatal hypoglycemia had no impact [29]. More studies are needed on sex-differential adverse outcomes in perinatal risk groups and neonates with hypoglycemia.

Lastly, our adjusted analysis identified an independent seemingly U-shaped association between maternal educational level and IQ score of the children. Parental educational level, or income, is routinely considered markers for IQ, which shows strong correlations with offspring IQ scores [30, 31]. Contrasting higher offspring IQ for mothers with the lowest education may be explained by ongoing education, or increased stimulation of verbal skills by home-going or short-time working mothers [32, 33].

Limitations of our study included the observational, retrospective design and the relatively small cohort size which was underpowered to detect a difference in IQ score difference of 3. Differences in secondary outcomes were subjected to risk of chance findings from multiple testing. Moreover, the non-participant rate was 62% (116/187 of the eligible children). However, included participants had parents of all educational levels, suggesting representativeness from the background population. We did not consider age difference between the hypoglycemia group and their siblings as a limitation, as all outcome score values were standardized for age.

Strengths included hospital file review of the neonatal hypoglycemia data, follow-up data on subsequent brain injuries or diseases that would meet exclusion criteria, the use of healthy siblings as controls, the detailed outcome testing, and adjustment for potential confounders, including parental educational level.

## Conclusion

In neonates without other severe risk factors for neurodevelopmental impairment but hypoglycemia < 1.7 mmol/L treated with > 2.5 mmol/L, we only detected a lower fine motor function within the normal range at follow-up, especially in boys. No changes in cognitive function or behavior were found. More and larger studies on sex-differential neurodevelopmental outcome in neonates with hypoglycemia and perinatal risk factors are needed.

## Data Availability

Data are available on request.

## Abbreviations

CBCL: Child Behavior Checklist
GA: Gestational age
LGA: Large for gestational age
Movement ABC-2: Movement Assessment Battery for Children 2
SGA: Small for gestational age
WISC-IV: Wechsler’s intelligence scale for children fourth edition

## References

[1] Hawdon JM (2013). Definition of neonatal hypoglycaemia: time for a rethink?. Arch Dis Child Fetal Neonatal Ed.

[2] Stanley CA, Rozance PJ, Thornton PS, De Leon DD, Harris D, Haymond MW, Hussain K, Levitsky LL, Murad MH, Simmons RA (2015). Re-evaluating “transitional neonatal hypoglycemia”: mechanism and implications for management. J Pediatr.

[3] Burns CM, Rutherford MA, Boardman JP, Cowan FM (2008). Patterns of cerebral injury and neurodevelopmental outcomes after symptomatic neonatal hypoglycemia. Pediatrics.

[4] Rozance PJ, Hay WW (2006). Hypoglycemia in newborn infants: features associated with adverse outcomes. Biol Neonate.

[5] Cornblath M, Reisner SH (1965). Blood glucose in the neonate and its clinical significance. N Engl J Med.

[6] Cornblath M, Hawdon JM, Williams AF, Aynsley-Green A, Ward-Platt MP, Schwartz R, Kalhan SC (2000). Controversies regarding definition of neonatal hypoglycemia: suggested operational thresholds. Pediatrics.

[7] Hawdon JM, Ward Platt MP, Aynsley-Green A (1992). Patterns of metabolic adaptation for preterm and term infants in the first neonatal week. Arch Dis Child.

[8] Altman M, Vanpee M, Cnattingius S, Norman M (2011). Neonatal morbidity in moderately preterm infants: a Swedish national population-based study. J Pediatr.

[9] Agrawal RK, Lui K, Gupta JM (2000). Neonatal hypoglycaemia in infants of diabetic mothers. J Paediatr Child Health.

[10] Maayan-Metzger A, Lubin D, Kuint J (2009). Hypoglycemia rates in the first days of life among term infants born to diabetic mothers. Neonatology.

[11] Adamkin DH (2011). Postnatal glucose homeostasis in late-preterm and term infants. Pediatrics.

[12] Harris DL, Weston PJ, Harding JE (2012). Incidence of neonatal hypoglycemia in babies identified as at risk. J Pediatr.

[13] Wechsler D (2003) Wechsler intelligence scale for children. Fourth edition (WISC-IV). San Antonio, TX: The Psychological Corporation

[14] Achenbach TM, Edelbrock CS (1981). Behavioral problems and competencies reported by parents of normal and disturbed children aged four through sixteen. Monogr Soc Res Child Dev.

[15] Henriksen J, Nielsen PF, Bilenberg N (2012). New Danish standardization of the Child Behaviour Checklist. Dan Med J.

[16] Kerstjens JM, Bocca-Tjeertes IF, de Winter AF, Reijneveld SA, Bos AF (2012). Neonatal morbidities and developmental delay in moderately preterm-born children. Pediatrics.

[17] (2004) Screening guidelines for newborns at risk for low blood glucose. Paediatr Child Health 9:723–740 10.1093/pch/9.10.723 PMC2724150 19688086

[18] Van Kempen AAMW, Eskes PF, Nuytemans DHGM, van der Lee J, Dijkman LM, van Veenendaal NR, et al. (2020) Lower versus traditional treatment threshold for neonatal hypoglycemia. N Engl J Med 2020;382(6):534–544 10.1056/NEJMoa1905593 32023373

[19] McKinlay CJD, Alsweiler JM, Anstice NS, Burakevych N, Chakraborty A, Chase JG, Gamble GD, Harris DL, Jacobs RJ, Jiang Y, Paudel N, et al. (2017) Association of neonatal glycemia with neurodevelopmental outcomes at 4.5 years. JAMA Pediatr 2017;171(10):972–983 10.1001/jamapediatrics.2017.1579 PMC5710616 28783802

[20] Schulz J, Henderson SE, Sugden DA, Barnett AL (2011). Structural validity of the Movement ABC-2 test: factor structure comparisons across three age groups. Res Dev Disabil.

[21] Harris DL, Weston PJ, Signal M, Chase JG, Harding JE (2013). Dextrose gel for neonatal hypoglycaemia (the sugar babies study): a randomised, double-blind, placebo-controlled trial. Lancet.

[22] Harris DL, Alsweiler JM, Ansell JM, Gamble GD, Thompson B, Wouldes TA, Yu TY, Harding JE (2016). Outcome at 2 years after dextrose gel treatment for neonatal hypoglycemia: follow-up of a randomized trial. J Pediatr.

[23] McKinlay CJ, Alsweiler JM, Ansell JM, Anstice NS, Chase JG, Gamble GD, Harris DL, Jacobs RJ, Jiang Y, Paudel N (2015). Neonatal glycemia and neurodevelopmental outcomes at 2 years. N Engl J Med.

[24] Boardman JP, Wusthoff CJ, Cowan FM (2013). Hypoglycaemia and neonatal brain injury. Arch Dis Child Educ Pract Ed.

[25] Boluyt N, van Kempen A, Offringa M (2006). Neurodevelopment after neonatal hypoglycemia: a systematic review and design of an optimal future study. Pediatrics.

[26] Kaiser JR, Bai S, Gibson N, Holland G, Lin TM, Swearingen CJ, Mehl JK, ElHassan NO (2015). Association between transient newborn hypoglycemia and fourth-grade achievement test proficiency: a population-based study. JAMA Pediatr.

[27] Lahat A, van Lieshout RJ, Mathewson KJ, Mackillop J, Saigal S, Morrison KM, Boyle MH, Schmidt LA (2017). Extremely low birth weight babies grown up: gene-environment interaction predicts internalizing problems in the third and fourth decades of life. Dev Psychopathol.

[28] Baron IS, Erickson K, Ahronovich MD, Baker R, Litman FR (2011). Cognitive deficit in preschoolers born late-preterm. Early Hum Dev.

[29] Clausen TD, Mortensen EL, Schmidt L, Mathiesen ER, Hansen T, Jensen DM, Damm P (2013). Cognitive function in adult offspring of women with gestational diabetes-the role of glucose and other factors. PLoS One.

[30] Rizzo TA, Metzger BE, Dooley SL, Cho NH (1997). Early malnutrition and child neurobehavioral development: insights from the study of children of diabetic mothers. Child Dev.

[31] Scarr S (1981). Race, social class, and individual differences in IQ.

[32] Scarr S (1992). Developmental theories for the 1900s: developmental and individual differences. Child Dev.

[33] Persson B, Gentz J (1984). Follow-up of children of insulin-dependent and gestational diabetic mothers. Neuropsychological outcome. Acta Paediatr Scand.

